# Late Life Divorce and Emotional Experiences That Follow: An Intergenerational Family Perspective

**DOI:** 10.1093/geroni/igaf122.1760

**Published:** 2025-12-31

**Authors:** Chaya Koren, Yafit Cohen, Naor Demeter

**Affiliations:** University of Haifa, Haifa, HaZafon, Israel; University of Haifa, Haifa, HaZafon, Israel

## Abstract

Late life divorce is rising in Western societies, and accordingly, more scientific knowledge is required regarding emotional experiences of being alone following late life divorce. Our aim was to examine emotional experiences following late life divorce from an intergenerational family perspective, considering late-life divorce consequences for the wellbeing of ex-spouses and their adult children. Data was collected through 51 semi-structured qualitative, in-depth interviews, comprised of 7 family units (n = 33) and 9 parent-child dyads (n = 18), using principles of thematic-analysis and dyadic interview-analysis. Findings reveal emotions related to loneliness and freedom. Different ex-family members’ perceptions regarding these experiences were identified. Most ex-spouses were willing to pay the price of loneliness to gain freedom. However, their adult-children mainly described the disadvantages of late-life divorce, emphasizing loneliness, and perceiving both loneliness and freedom as negative. These gaps could be explained in two ways: (1) The adult children see their parents in their parental role and less as individuals enjoying freedom outside of the family context following late life divorce. Therefore, their adult children have difficulty seeing the benefits of freedom. (2) The adult children feel responsibility to relief their parents’ loneliness, which causes them an emotional burden. Unique aspects of freedom and loneliness in old age along with coping strategies of both generations are discussed. Implications for families and family professionals are addressed.

